# Characterizing the heterogeneous correlations between the landscape patterns and seasonal variations of total nitrogen and total phosphorus in a peri-urban watershed

**DOI:** 10.1007/s11356-020-09441-5

**Published:** 2020-06-15

**Authors:** Chongwei Li, Haiyan Zhang, Yonghong Hao, Ming Zhang

**Affiliations:** 1 School of Geographic and Environmental Sciences, Tianjin Normal University, Tianjin, 300387 China; 2 Tianjin Key Laboratory of Water Resources and Environment, Tianjin Normal University, Tianjin, 300387 China; 3 Geological Survey of Japan, AIST, Tsukuba, Ibaraki, 305-8567 Japan

**Keywords:** Nitrogen and phosphorus pollution, Landscape patterns, Geographical detector, Geographically weighted regression, Integrated effects

## Abstract

Landscape patterns in a watershed potentially have significant influence on the occurrence, migration, and transformation of pollutants, such as nitrogen (N) and phosphorus (P) in rivers. Human activities can accelerate the pollution and complicate the problem especially in a peri-urban watershed with different types of land use. To characterize the heterogeneous correlations between landscape patterns and seasonal variations of N and P in a peri-urban watershed located upstream of Tianjin metropolis, China, observations of total nitrogen (TN) and total phosphorus (TP) at 33 locations were performed in the wet and dry seasons from 2013 to 2016. The data from individual locations were averaged for the wet and dry seasons and analyzed with geographical detector to identify influential landscape indices on seasonal water quality variations. The geographically weighted regression method, capable of analyzing heterogeneous correlations, was used to evaluate the integrated effects from different landscape indices. The results demonstrated that the location-weighted landscape contrast index (LWLI), the ratio of urban areas, and the ratio of forest areas were major influential indicators that affected TN and TP in river water. These indices also had integrated effects on variations of TN and TP together with other indices such as Shannon diversity index, landscape shape index, largest patch index, and contagion index. The integrated effects were different in the wet and dry seasons because of different effects of flushing and dilution by rainwater and the heterogeneity in landscape patterns. The LWLI had a positive relationship to water quality in the areas with high ratio of urban areas, indicating that domestic wastewater can be a major source of N and P pollution. The approaches and findings of this study may provide a reference for characterizing the major factors and integrated effects that control nonpoint source pollution in a watershed.

## Introduction

A wide range of human activities may cause discharge of nitrogen (N) and phosphorus (P) into water environments, resulting in eutrophication of the water bodies and deterioration of water quality in rivers (Bu et al. 2014; Liu et al. 2018a). Landscape patterns in a peri-urban watershed, an interaction zone with adjacent urban and rural activities, may undergo modifications by human activities (Li et al. 2018; Lantz et al. 2013). Rivers are an important component of a watershed landscape, and changes in river water quality are closely related to landscape patterns. (Mello et al. 2018). The landscape pattern in a catchment can be characterized by landscape types, or types of land use and structure, and has a significant impact on the hydro-ecological processes within it. Quantitative landscape indices reflect the landscape types and structure, and can be used to comprehensively evaluate the effects of landscape patterns on water quality in a catchment (e.g., Uuemaa et al. 2007; Xu et al. 2019a). Some landscape indicators could predict the changes in water quality and be used to control and manage water quality in a watershed (Uuemaa et al. 2007; Xu et al. 2019a). Although several case studies on relationships between landscape patterns and water quality in catchments are available (Tu 2013; Han et al. 2014; Chen et al. 2016), most of them are focused on landscape composition and structure, such as the percentages of land use type and the impacts of landscape indices on water quality. Because of the complexity of water body pollution processes, approaches that analyze the spatial distribution and integrated effects of different areas and landscape indicators on water quality remain to be explored (Wu and Lu 2019).

Statistical analyses such as correlation analysis, principal component analysis, and conventional linear regression can be used to analyze the effects of landscape patterns on river water pollution. In addition, multivariate stepwise regression and redundancy analysis can also be used to evaluate the factors that cause seasonal variations in river water quality (Yu et al. 2016; Wu and Lu 2019; Xu et al. 2019b). However, all these methods are based on global statistics so that spatial changes in influential factors are difficult to consider. The spatial heterogeneity may have significant influence on nitrogen and phosphorus pollution, but this topic has not been addressed so far for a watershed and cannot be evaluated with traditional linear statistical approaches. The objectives of this study were twofold: 1) to identify influential landscape indices that may affect seasonal variations of water quality in rivers and 2) to evaluate heterogeneous correlations between landscape patterns and water quality in rivers caused by the integrated effects of different landscape indices. To realize these objectives, the geographical detector method, a novel method based on spatial variance analysis (Wang et al. 2010, 2016), and the geographically weighted regression method (Fotheringham et al. 2002; Robinson et al. 2013; Chu et al. 2018), which can evaluate spatial variations in the relationships between dependent and independent variables, were adopted for the first and second objectives, respectively. The methods were then used to analyze the data associated with total nitrogen (TN) and total phosphorus (TP) measured in river water at 33 points from 2013 to 2016 in the Yuqiao watershed. This watershed contains the Yuqiao reservoir, the only one water supply source for Tianjin city, the third largest municipality of China, and with a population over 15 million (Wu et al. 2019; Wen et al. 2019). The study on water quality in Yuqiao watershed is very important for the security of water resource for Tianjin Municipality.

## Study area

**Fig. 1:**
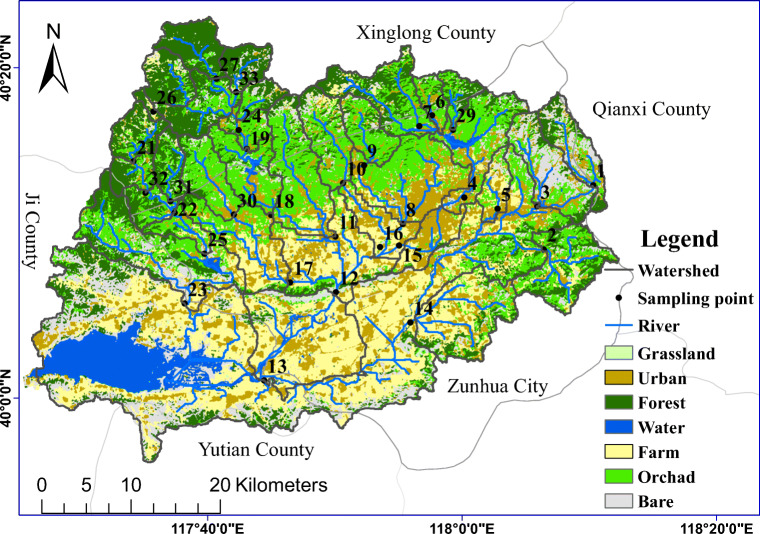
The location of and land use in Yuqiao watershed (modified from Zhang et al. 2017)

We selected the Yuqiao watershed as the study area because previous fundamental study based on stepwise regression and redundant analysis illustrated that the watershed contains complicated landscape structures (Zhang et al. 2017), which can be preferable for the study on spatial heterogeneity. The Yuqiao watershed extends across 39° 56′ N–40° 23′ N and 117° 26′ E–118° 12′ E and has a total area about 2060 km^2^ (Fig. 1). It is located about 4 km east of Jizhou District in the north of Tianjin municipality, in the southern part of Hebei Province, China. The terrain descends from northeast to southeast, with mountains in the northwest, plains in the middle, and hills and low mountains in the south parts. Major soil types within the watershed are brown and cinnamon soils. The location belongs to a warm, continental monsoon region, with a semi-humid climate and an average annual precipitation greater than 700 mm. The average temperatures in the dry (April to June) and wet (July to September) seasons were about 19 °C and 24.5 °C, respectively, during the years 2013–2016. According to the data measured at one of the weather stations established within the Yuqiao watershed, the accumulated precipitations during the dry season were about 130 mm, 270 mm, 180 mm, and 200 mm, and the accumulated precipitations during the wet season were about 550 mm, 375 mm, 320 mm, and 347 mm in each year between 2013 and 2016. Major rivers that flow into the basin are the Sha, Lin, and Li rivers. Villages and towns are widely distributed in the basin with a population about 800,000, and various types of pollution may affect the water quality in the rivers running across the watershed (Wen et al. 2019). To analyze the water environment within the watershed in detail, the basin catchment area was divided into 33 sub-basins using the hydrology toolset in ArcGIS and 1:50,000 DEM. To avoid potential influence of the water diversion project, divisions of sub-basin were concentrated in the upstream and middle areas of the basin.

**Fig. 2:**
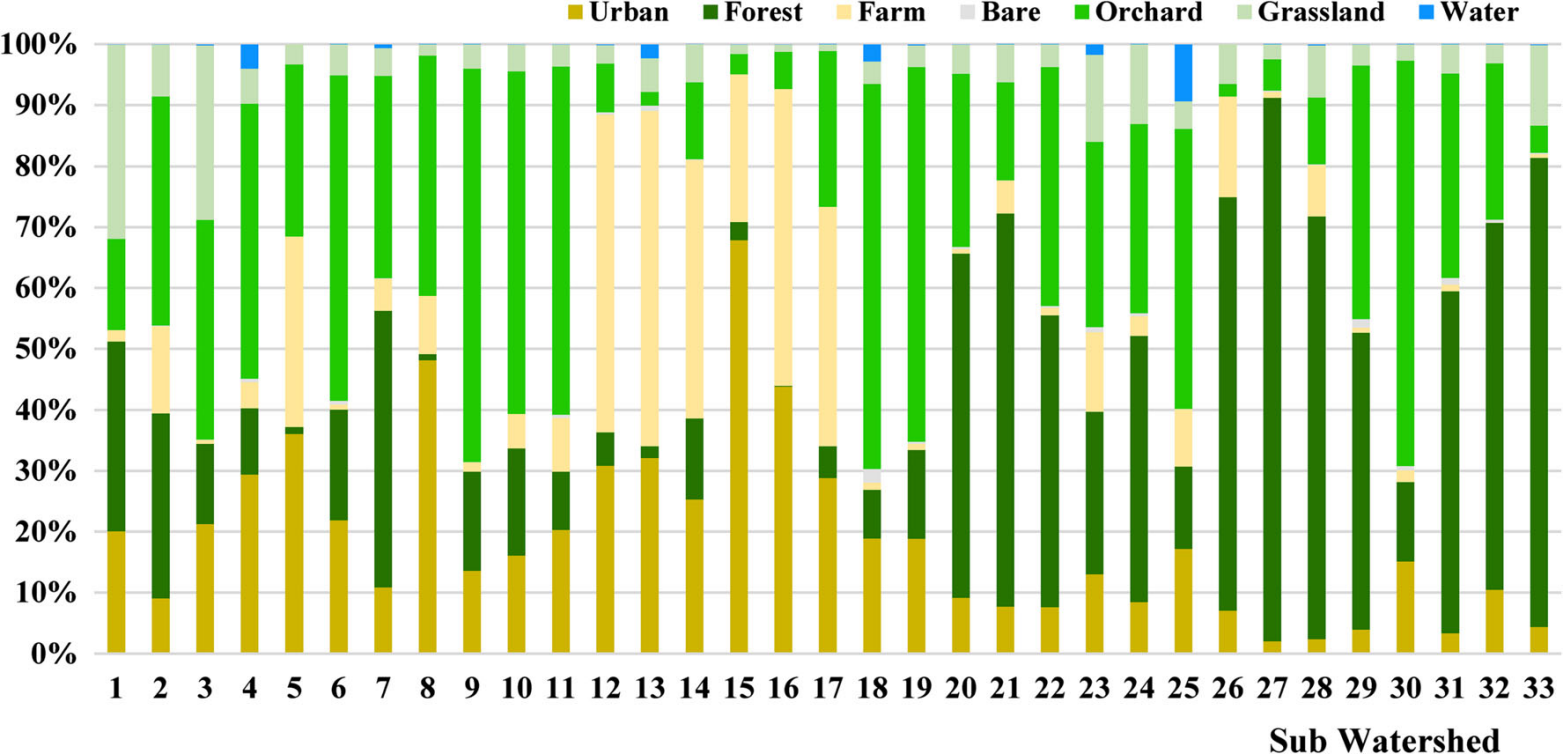
Composition of land use types in individual sub-basins in Yuqiao watershed (updated from Zhang et al. 2017)

The watershed mainly consists of forest, urban, orchard, bare, grass, and farm lands (Fig. 1). Landscape patterns were unchanged from those reported in Zhang et al. (2017), which were analyzed with the Landsat 8 remote sensing image taken in 2013. In this study, the land use data were extracted and interpreted from the Landsat 8 remote sensing image taken in August 2015. The types of land use in each sub-basin were characterized with the same approach used in Zhang et al. (2017), and shown in Fig. 2.

## Methods

### Sampling and water quality analysis

Sampling of river water was performed at the downstream point of each sub-basin (a total of 33 sub-basins; Fig. 1) in the dry (April to June) and wet (July to September) seasons from 2013 to 2016. Sampling during the dry season was basically performed every month in the middle of month, and sampling during the wet season was performed after moderate rains (> 10 mm/24 h) and heavy rains (> 25 mm/24 h). A total of 957 samples were collected and analyzed over the 4-year period. The sampling procedures were conducted in accordance with the “technical specifications requirements for monitoring of surface water and wastewater” issued by the China’s State Environmental Protection Administration (HJ-T91-2002). We took 500 ml of water from each point, sealed it within a glass bottle, and kept the temperature below 4 °C during transportation and storage before laboratory analyses. The study objectives were to characterize the heterogeneous correlations between the landscape patterns and seasonal variations in river water quality, so TN and TP were selected as indicators for pollution, although both nitrogen and phosphorus have different forms in natural conditions. TN and TP in water samples were analyzed with the alkaline potassium persulfate digestion UV spectrophotometric method and ammonium molybdate spectrophotometric method described by Chinese GB11894–89 and GB11893–89, respectively, with reference to the GB3838-2002 on environmental quality standards for surface water.

### Landscape metric analysis

Landscape metrics were calculated with FRAGSTATS (version 4.2), a commercialized software that is designed to compute a wide variety of landscape metrics for categorical map patterns. Six landscape indices, specifically Shannon’s diversity index (SHDI), contagion index (CON), landscape shape index (LSI), edge density (ED), largest patch index (LPI), and patch density (PD) were selected for the computation. Table 1 briefly summarizes the functions and roles of the individual indicators (Peng et al. 2010). In addition, the location-weighted landscape contrast index (LWLI), a scale-independent approach for landscape pattern evaluation based on source-sink ecological processes, was also used. This index was calculated using Eqs. 1 and 2 (Chen et al. 2009):

**Table 1:** The functions and roles of major indices describing landscape patterns

Index	Function and role
Shannon’s diversity index (SHDI)	Reflects the diversity or heterogeneity of landscapes
Contagion index (CON)	Reflects the observed contagion over the maximum possible contagion for the given number of patch types
Landscape shape index (LSI)	Reflects the complexity of landscape patch shapes and provides a measure of class aggregation
Edge density (ED)	Reflects the degree of landscape fragmentation
Largest patch index (LPI)	Quantifies the percentage of total landscape area comprised by the largest patch at the class level
Patch density (PD)	Quantifies the number of patches per unit area

1 $$ {\mathrm{LWLI}}^{\prime }={\sum}_{i=1}^m{A}_i\times {W}_i\times {AP}_i\div \left[{\sum}_{i=1}^m{A}_i\times {W}_i\times {AP}_i+{\sum}_{j=1}^n{S}_j\times {W}_j\times {AP}_j\right] $$

2 $$ \mathrm{LWLI}={\mathrm{LWLI}}^{\prime}\mathrm{distance}\times {\mathrm{LWLI}}^{\prime}\mathrm{elevation}\div {\mathrm{LWLI}}^{\prime}\mathrm{slope} $$

where *A*_*i*_ and *S*_*j*_ refer to the areas of the *i*th “source” and *j*th “sink” landscapes; *W*_*i*_ and *W*_*j*_ are the weights for the “source” and “sink” landscapes; *AP*_*i*_ and *AP*_*j*_ refer to the percentages of the *i*-source and *j*-type landscapes; and *m* and *n* are the numbers of “source” and “sink” landscape types, respectively. LWLI^’^_distance_, LWLI^’^_elevation_, and LWLI^’^_slope_ are the values of LWLI with respect to the distance, relative elevation, and slope gradient, respectively. We categorized forest and grassland as “sink” landscapes while farm, urban, and orchard lands as “source” landscapes. The weights for the forest, grassland, farmland, urban land, orchard land, and bare land were assigned to be 0.8, 0.6, 0.4, 1.0, 0.4, and 0.5, respectively, with reference to Chen et al. (2009).

### Geographical detector

Geographical detector is a novel tool originally proposed by Wang et al. (2010, 2016) for investigating spatial stratified heterogeneity. The basic principle of geographical detector is to compare the spatial consistency of driving forces (e.g., the types of land use and landscape indices) with relevant resultant outcomes (Wang et al. 2010) such as water quality (TN and TP in river water in this study). This technique assumes that a risk exhibits a similar distribution as the factors that lead to the risk. The power of the determinant value, corresponding to the significance of effects of a landscape index or land use type in this study, for the geographical detector, is defined in Eq. 3 (Wang et al. 2016):

3 $$ q=1-\frac{\sum \limits_{\mathrm{h}=1}^{\mathrm{L}}{\sum}_{\mathrm{i}}^{{\mathrm{N}}_{\mathrm{h}}}{\left({\mathrm{Y}}_{\mathrm{h}\mathrm{i}}-\overline{{\mathrm{Y}}_{\mathrm{h}}}\right)}^2}{\sum \limits_{\mathrm{i}}^{\mathrm{N}}{\left({\mathrm{Y}}_{\mathrm{i}}-\overline{\mathrm{Y}}\right)}^2}=1-\frac{\sum \limits_{\mathrm{h}=1}^{\mathrm{L}}{\mathrm{N}}_{\mathrm{h}}{\upsigma}_{\mathrm{h}}^2}{\mathrm{N}{\upsigma}^2}=1-\frac{\mathrm{SSW}}{\mathrm{SST}} $$

where *h* = (1 to L) is the number of classification; *N*_*h*_ and *N* are the number of sampling units in layer *h* and the whole region, respectively; and*σ*_*h*_ and *σ* are the variances of the layer *h* and whole region, respectively. SST and SSW are the total sum of squares and within sum of squares, respectively. The *q*-statistic is a monotonic function of the strength of the spatial stratified heterogeneity and *q*∈[0, 1]. It increases as the strength of the stratified heterogeneity increases, meaning that a larger *q* value corresponds to more significant effects of a landscape index or land use type on water quality.

The most important advantage of geographical detector over traditional approaches is that there are less assumptions and less constraints. The tool can be used to filter out and differentiate the relative importance of determinants based on spatial variation (Liu et al. 2018b). This method has recently been used to analyze the factors affecting the loss of phosphorus from soils (Liu and Wang et al. 2018) and the sources for heavy metal pollution in basins (Luo et al. 2019).

The interaction detector in the geographical detector method can be used to examine whether two (or more) factors (*X*_1_, *X*_2_) have an interactive influence on a response variable through comparison of *q* values (Zuo et al. 2018; Luo et al. 2019). A description of the interactive relationships between two factors is tabulated in Table 2.

**Table 2:** Types of interactive relationships between two factors defined in geographical detector

Description	Interaction
*q*(*X*1∩*X*2) < Min(*q*(*X*1),*q*(*X*2))	Weakened, nonlinear
Min(*q*(*X*1),*q*(*X*2)) < *q*(*X*1∩*X*2) < Max(*q*(*X*1),*q*(*X*2))	Weakened, single factor nonlinear
*q*(*X*1∩*X*2) > Max(*q*(*X*1),*q*(*X*2))	Enhanced, double factors
*q*(*X*1∩*X*2) = *q*(*X*1)+ *q*(*X*2)	Independent
*q*(*X*1∩*X*2) > *q*(*X*1)+ *q*(*X*2)	Enhanced, nonlinear

### Geographically weighted regression

Geographically weighted regression is an extension of traditional least squares regression that allows a model, i.e., the relationships between dependent and independent variables, to vary over space so that spatial heterogeneity can be considered (Fotheringham et al. 2002). The regression coefficient *β* is no longer a global constant but becomes a dependent constant, *β*_*i*_, to the spatial position. The regression models that underlie geographically weighted regression are expressed in Eq. 4:

4 $$ {y}_{\mathrm{i}}={\beta}_0\left({u}_i,{v}_i\right)+\sum \limits_{k=1}^p{\beta}_k\left({u}_i,{v}_i\right){x}_{ik}+{\varepsilon}_i $$

where *x*_*i*_ and *y*_*i*_ are the independent and dependent variables, respectively; (*u*_*i*_, *v*_*i*_) is the coordinate of *i*th sampling point; β_k_ (*u*_*i*_, *ν*_*i*_) is the *k*th regression coefficient obtained for the sampling point *i*; and β_0_ (*u*_*i*_, *ν*_*i*_) and *ε*_*i*_ are the intercept and residual obtained from the model for the sampling point *i*, respectively.

The regression coefficient, β_k_ (*u*_*i*_, *ν*_*i*_), can be estimated using the method of weighted least squares with the weights defined with Eq. 5:

5 $$ {w}_{ij}=\exp \left[-\frac{1}{2}{\left(\raisebox{1ex}{${d}_{ij}$}\!\left/ \!\raisebox{-1ex}{$b$}\right.\right)}^2\right] $$

where *w*_*ij*_ is the weight given to data point *j* for the estimation of local parameters at point *i*; *d*_*ij*_ is the distance between the points *i* and *j*; and *b*, named kernel bandwidth, is a non-negative attenuation function describing the relationship between the weight and distance (Purwaningsih and Erfiani 2015). A fixed value of *b* was adopted and was determined by minimizing Akaike’s information criterion in this study (da Silva and Mendes 2018).

Geographically weighted regression has been applied to study the effects of landscape structure, such as the types of land use (Tu 2013; Chen et al. 2016) and population density (Chen et al. 2016) on regional water quality. Tu (2013) concluded that geographically weighted regression has better model performance than ordinary least squares regression. The effects of landscape pattern indices, however, were not examined in their studies. In general, geographically weighted regression is limited by collinearity among variables, and the significance of collinearity increases with an increase in the number of variables. To avoid this limitation, we used geographical detector to filter out the landscape indices with *q* < 0.5 that do not have significant effects on the river water quality and to reduce the number of variables before the geographically weighted regression analysis.

After the geographically weighted regression, the *t* values for local parameter estimates were further determined through *t* tests, because the *t* test can be used to analyze the significance of parameter estimates as an effective exploratory tool (Malczewski and Poetz 2005; Jaimes et al. 2010; Tu 2013). If *t* > 0, it means that the influential factor (i.e., a landscape index or a type of land use) has a positive relationship with the result (i.e., water quality in this study). If *t* < 0, the relationship becomes negative. The bigger the absolute value of *t*, the more significant the effect of landscape index or the type of land use will be on river water quality. The *p* value can be determined from the *t* value and its degree of freedom. When the significance level *α* = 0.01, the corresponding *t* value equals ± 2.59; when *α* = 0.05, the corresponding *t* value equals ± 1.97; and when *α* = 0.1, the corresponding *t* value equals ± 1.65.

## Results

### Water quality

**Fig. 3:**
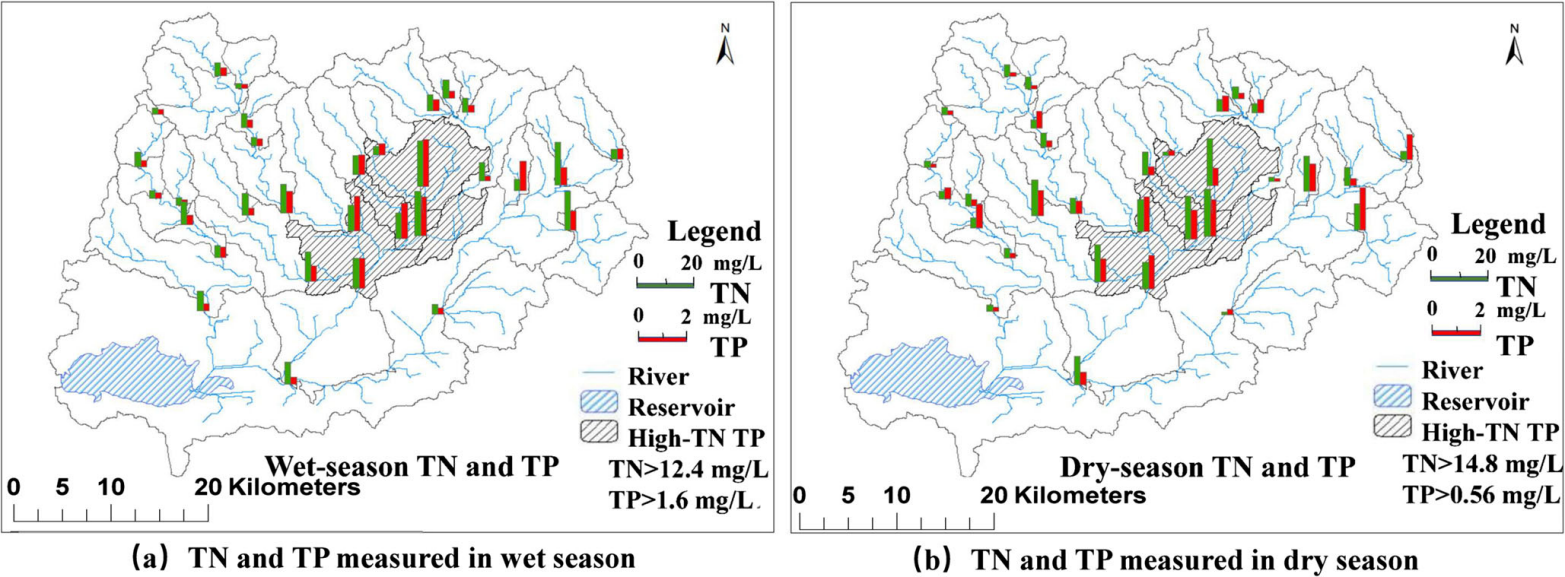
The average concentrations of TN and TP measured at each sampling point in wet and dry seasons in Yuqiao watershed from 2013 to 2016

The TN and TP in river water collected at each of the 33 sampling points during the wet and dry seasons between 2013 and 2015 did not show significant variations (Zhang et al. 2017). The values were averaged with the water quality data from 2016 and depicted in Fig. 3 a and b. The TN ranged from 2.10 to 18.80 mg/L and 1.16 to 22.00 mg/L in the wet and dry seasons, respectively, and the TP ranged from 0.20 to 1.96 mg/L and 0.04 to 0.56 mg/L in the wet and dry seasons, respectively. Note that TN and TP are shown on different scales because the TN concentration is several times higher than the TP concentration. The concentrations of TN and TP have significant spatial heterogeneity.

### Effects and integrated effects of landscape patterns on river water quality

**Fig. 4:**
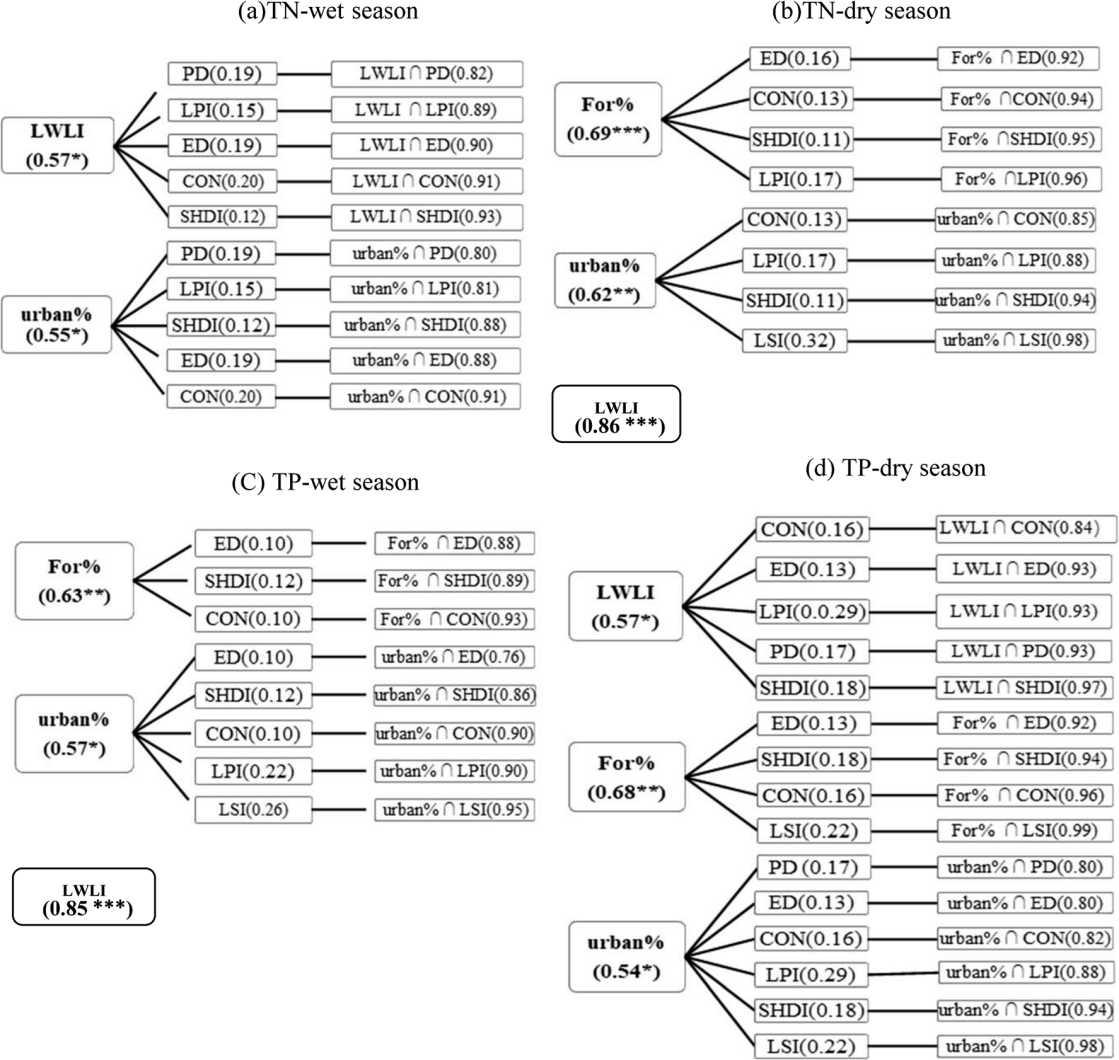
The values of *q* estimated independently for each landscape indicator (1st and 2nd columns) and under combination of two landscape indicators (3rd column). *, **, and *** indicate that the *p* values with significance levels *α* = 0.05, 0.01, and < 0.001, respectively. LWLI, Urban%, and For% denote the location-weighted landscape contrast index, ratio of urban areas, and ratio of forest areas, respectively. PD denotes patch density, LPI denotes the largest patch index, ED denotes edge density, CON denotes contagion index, SHDI denotes Shannon’s diversity index, and LSI denotes landscape shape index, respectively

The value of *q* reflects the significance of the relationships between each landscape index or the type of land use and river water quality in the wet and dry seasons, estimated by the geographical detector (Fig. 4). The first column states the factors (i.e., landscape index and land use type) that have significant effects (*q* > 0.5). The second column states the factors that affect river water quality but are not significant (*q* < 0.5). The third column states the integrated effects of the two factors listed in the first and second columns.

### Factors that had strong positive effects on river water quality

**Fig. 5:**
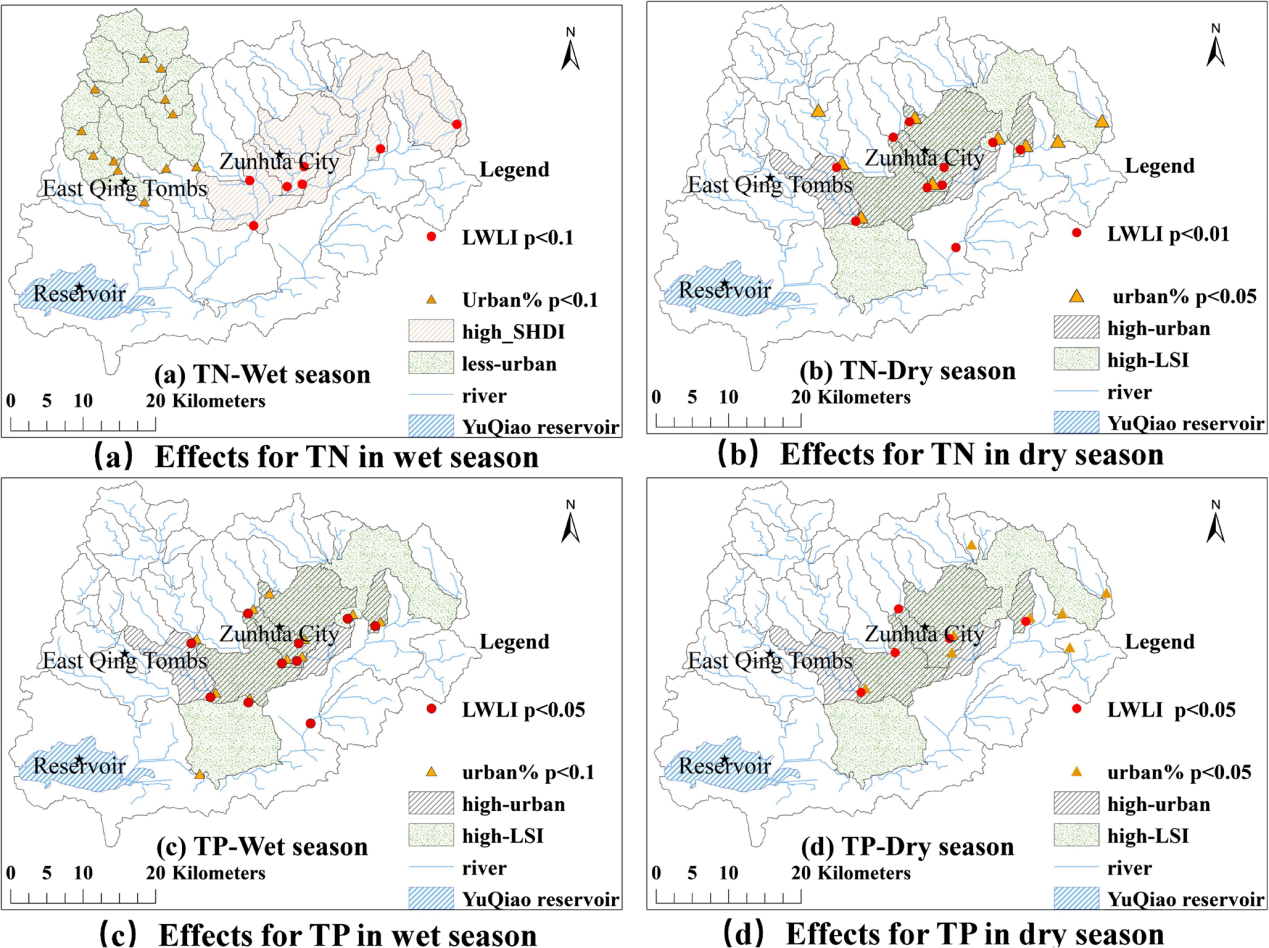
Landscape indicators that had strong positive effects on river water quality (sub-basins passed *p* < 0.1 in *t* tests)

A positive *t* value obtained from the *t* test corresponds to positive effects, and the value of *p* obtained from the *t* test can be used to evaluate the significance of the effects of a landscape pattern indicator (either landscape index or land use type) on river water quality. Landscape indicators that had strong positive effects on TN and TP in river water are shown in Fig. 5. The sub-basins that had the effects from LWLI (*p* < 0.1 and *p* < 0.01) and from Urban% on TN (*p* < 0.1 and *p* < 0.05) in wet and dry seasons are shown in Fig. 5 a and b, respectively. The sub-basins that showed the effects from LWLI (*p* < 0.05) and Urban% on TP (*p* < 0.1 and *p* < 0.05) in wet and dry seasons are shown in Fig. 5 c and d, respectively. TN and TP concentrations followed different trends, which were also different between wet and dry seasons, as the plots showing significant effects from the landscape indices (i.e., the LWLI and Urban%) are located in different sub-basins and have different significance levels (*p* values).

### Factors that had strong negative effects on river water quality

**Fig. 6.**
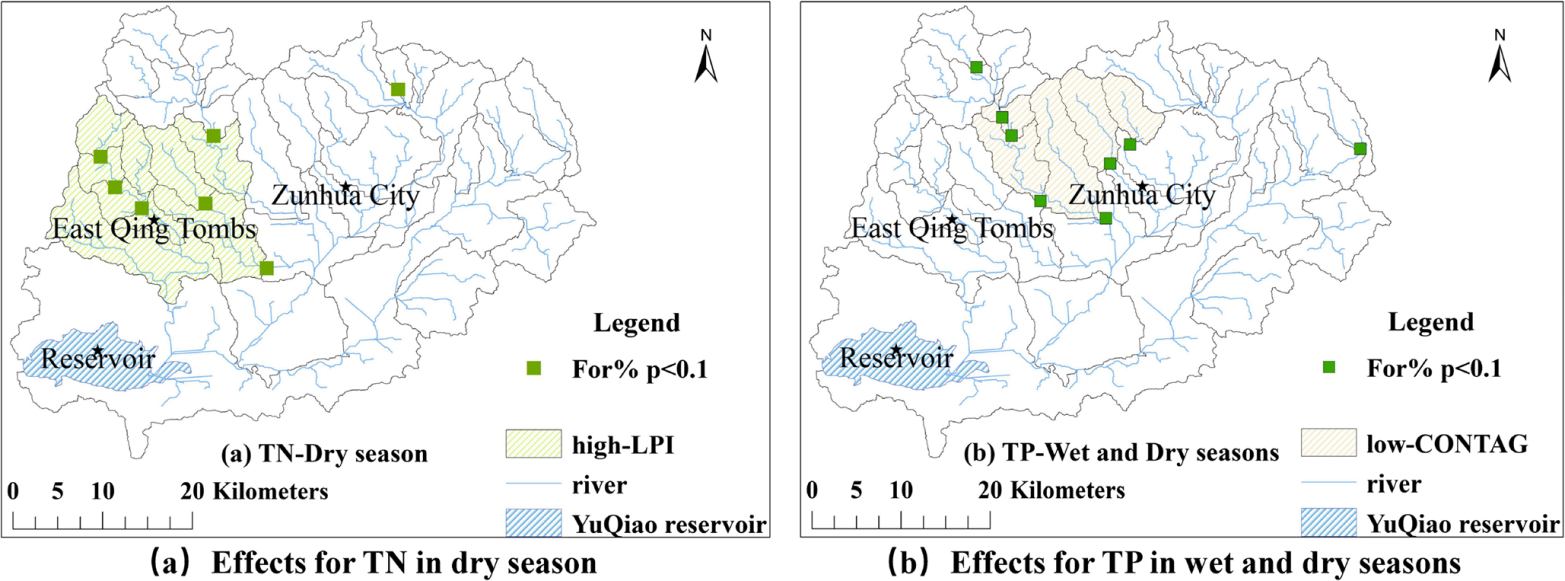
The ratio of forest (For%) that had strong negative effects on river water quality (sub-basins passed *p* < 0.1 in *t* tests)

A negative *t* value obtained from the *t* test corresponds to negative effects. The ratio of forest areas (For%) was characterized as the only factor that had strong negative effects on river water quality (Fig. 6), where Fig. 6a shows the sub-basins that had strong negative effects on TN (*p* < 0.1) in the dry season (but not for the wet season), and Fig. 6b shows the sub-basins that had strong negative effects on TP (*p* < 0.1) in both the wet and dry seasons.

## Discussion

### Spatial distribution and seasonal variations of TN and TP

The differences in concentrations of TN and TP varied between sampling points, indicating that the strong spatial heterogeneity and/or landscape patterns may have significant effects on the spatial distribution of TN and TP in river water (Fig. 3). Such spatially heterogeneous correlations cannot be simply analyzed with traditional statistical analyses based on global statistics. A method that considers spatial heterogeneity, such as the geographical detector and geographically weighted regression methods adopted in this study, should be used for the analysis of the relationships between landscape patterns and river water quality. Compared with N, the P concentration significantly decreased in river water in the dry season, suggesting that the ratio of particulate phosphorus to dissolved phosphorus is high, and that rainfall is a key factor controlling the migration of P in the watershed.

### Effects of individual landscape indicator on river water quality

Overall, a series of landscape indicators independently affected river water quality, with *q* values varying from 0.1 (ED and CON for TP in wet season) to 0.86 (LWLI for TN in dry season; the first two columns in Fig. 4). The LWLI, For%, and Urban% were strongly related to the TN and TP in river water both in wet and dry seasons (the first column in Fig. 4, *q* > 0.5). The tendencies associated with the effects of For% and Urban% were consistent with the findings reported by Chen et al. (2016), Tu (2011, 2013), and Xu et al. (2019a). Human activities are concentrated in urban areas, inducing the release of nitrogen and phosphorus to the environment. The forest can act as a filter to capture both nitrogen and phosphorus (Mello, et al. 2018).

The average *q* values of the three indicators for TN and TP were 0.65, 0.60, and 0.58, respectively. Among these three indicators, the LWLI had the strongest relationships (*p* < 0.05; the first column in Fig. 4, and Fig. 5) with TN and TP in river water. The LWLI is an integrated index that considers the landscape patterns, spatial distance, and geographical characteristics (Chen et al. 2009; Wang et al. 2018), and it was the most effective indicator for evaluating the effects of landscape pattern on river water quality in the study area.

A more detailed examination found that the relationships between the indicators and water quality were different in wet and dry seasons (Fig. 5). In the wet season, the relationships between the influential landscape indicators, especially the LWLI, with TP were stronger (*p* < 0.05) than those with TN (*p* < 0.1). The tendency was opposite in the dry season with *p* < 0.05 and *p* < 0.01 for TP and TN, respectively. In general, rainfall in the wet season flushes the nitrogen and phosphorus from soils into rivers. In addition, phosphorus exists in dissolved and particulate forms, and particulate phosphorus is usually higher than dissolved phosphorus. Particulate phosphorus can be washed into rivers by rainfall in the wet season, resulting in the increase in measured TP concentration in the water (Han et al. 2014). Nitrogen has different forms, such as nitrate, ammonium nitrogen, and organic nitrogen, among which nitrate is most easily lost to water. Compared with phosphorus, nitrogen is relatively soluble and the TN concentration in river water is relatively high. Rainwater can dilute nitrogen in rivers and decrease in TN concentration in the wet season. Wastewater and leaching from city rubbish are a major source of nitrogen and can result in higher nitrogen concentrations compared with phosphorus (Han et al. 2014). Similar results were also reported by Li et al. (2016) and Cui et al. (2018).

In the wet season, the effects of Urban% on TN were concentrated in the areas with low Urban% (Fig. 5a). In areas with a higher ratio of orchard land, rainwater flushes the nitrogen (especially nitrogen fertilizers) from the soils in orchard lands and increases the concentration of N in related sub-basins (Tu 2013). In the dry season, the areas that had higher effects from LWLI were concentrated in the areas with high Urban% (Fig. 5 b and d). In such areas of high human activity, leaching from livestock wastes increases soluble nitrogen and phosphorus in related sub-basins (Bu et al. 2014).

Although the effects were not significant (*q* < 0.5), other types of land use, such as the ratio of farm land areas and the ratio of grass land areas, and landscape indices such as the PD and LSI also showed co-relationships with the river water quality (the second column in Fig. 4). The *q* values for the wet (*q* = 0.26 for LSI for TP in wet season) and dry (*q* = 0.32 for LSI for TN in dry season) seasons were different, indicating that river water quality has seasonal variations and depends on land use and landscape patterns.

### Integrated effects of landscape indicators on river water quality

The estimated values of *q* under combined conditions (the third columns in Fig. 4) were greater than those estimated independently for individual landscape indicators (the first and second columns in Fig. 4). Many of them had enhanced, nonlinear interactions with *q*(*X*1∩*X*2) > *q*(*X*1) + *q*(*X*2) and enhanced, double factors interactions with *q*(*X*1∩*X*2) > Max(*q*(*X*1), *q*(*X*2) (refer to Table 2 for interaction definition). These results indicated that the two indicators were interactive and had integrated or enhanced effects on river water quality. The landscape indicators, such as SDHI, CON, LSI, ED, LPI, and PD had low, independent effects (*p* > 0.05) on river water quality (the second column in Fig. 4) but had enhanced effects when combined with an indicator that had significant effects on river water quality (the first column in Fig. 4). For example, the *q* value for describing the significance of the relationships between the LWLI and TN was 0.57 in the wet season but increased to 0.93 when combined with the SHDI having an independent, low *q* value of 0.12. Similar examples can be found from most of the combinations given in Fig. 4. These results indicated again that the independent use of landscape indices as the factors for evaluating their effects on river water quality is not sufficient. In other words, the evaluation of the integrated effects based on the geographical detector is much more effective.

In the wet season, SHDI showed enhanced effects on TN in the rivers located in sub-basins having high values of the LWLI (Fig. 5a), where human activities, land use, and heterogeneity of landscapes can induce integrated effects. The LSI had a positive effect on TN in the dry season and on TP in the wet and dry seasons (Fig. 5 b, c, and d), because more complex landscape fragments contribute more to the release of nitrogen and phosphorus (Xu et al. 2019a).

LPI had negative effects on TN in the dry season (Fig. 6a), because the forest and grass land with large areas can capture the nitrogen (Xu et al. 2019a). CON had negative effects on TP in both wet and dry seasons (Fig. 6b), consistent with reports by Xu et al. (2019b) and Guo et al. (2018).

### Significance of the *t* test

The use of *t* test enabled the determination of whether the effects of a landscape indicator on river water quality was positive or negative (*t* > 0 or *t* < 0), while simultaneously judging the significance of the effects based on *p* value (Fig. 5 and Fig. 6). The LWLI and the Urban% had strong positive effects (Fig. 5), while the ratio of forest, For%, had strong negative effects (Fig. 6). The results were consistent with the findings reported in previous studies (Chen et al. 2016; Tu 2011, 2013; Xu et al. 2019a). In addition, these results indicate that forests capture both nitrogen and phosphorus, and human activities (in urban areas and those involved in LWLI) are a major source of nitrogen and phosphorus pollution.

A detailed examination of the data revealed that in the wet season, the effects of LWLI and the Urban% on TN were concentrated in the areas having high SHDI and low Urban% (Fig.5a), whereas their effects on TP were concentrated in the areas having high LSI and high Urban% (Fig. 5c). In the dry season, the effects of LWLI and Urban% on both TN and TP were concentrated in almost the same areas having high Urban% and high LSI (Fig. 5 b and d).

The effects of For% on water quality showed spatial-temporal differences (Fig. 6). The effects of For% on TN were not significant (*p* > 0.1) in the wet season, whereas the effects were significant in the dry season (*p* < 0.1). The effects of For% on TP were significant both in the wet and dry seasons (*p* < 0.1). High values of *t* were concentrated in the area having high LSI. The integrated effects of For% and LSI were enhanced when combined. Negative effects of For% on TP were concentrated in the area having low CON, meaning that their integrated effects were significant. These results indicated the effectiveness of evaluating the integrated effects of combined landscape indicators based on geographically weighted regression.

## Conclusions

Landscape patterns potentially affect the quality of river water in a watershed. In this study, we examined the effects of different kinds of landscape indicators, specifically the types of land use and landscape indices capable of describing the landscape structure, on TN and TP in the rivers in Yuqiao watershed at Tianjin, China, using the methods of geographical detector and geographically weighted regression. The major conclusions drawn from this study can be summarized as follows:

1. Compared with the Urban%, For%, and other landscape indicators, LWLI was the most effective index for evaluating the effects of landscape patterns on river water quality in the study area.
2. Independent use of landscape indices, such as SHDI, CON, LSI, ED, LPI, and PD, was not effective for evaluating effects on river water quality. Their effects were nonlinearly enhanced when combined with an indicator having significant effects such as LWLI, Urban%, and For%. The use of geographical detector, which considers the integrated effects of two landscape indicators, was effective.
3. The effects and integrated effects of landscape pattern indicators on river water quality were more significant in the dry season than in the wet season, especially for TP, indicating that rainfall is a driving force for the migration of pollutants in the watershed. Flushing and dilution of soluble nitrogen and dissolved and particulate phosphorus, along with release of nitrogen and phosphorus from human activities and capture by the forest, were the major mechanisms for dynamic change in the watershed.
4. The use of *t* tests enabled simultaneous evaluation of either positive or negative effects and the significance levels of the effect. Simultaneously using *t* test for detailed examination of the effects of landscape patterns on river water quality in the watershed was effective.

The approaches and findings of this study provide a method for finding critical source areas with significant effects on nonpoint source pollution in a watershed considering spatial heterogeneity. Further studies, such as on the correlations between landscape patterns and the existing forms of N and P, will be performed in the future.
